# Controlling Directionality and Dimensionality of Radiation by Perturbing Separable Bound States in the Continuum

**DOI:** 10.1038/srep33394

**Published:** 2016-09-19

**Authors:** Nicholas Rivera, Chia Wei Hsu, Bo Zhen, Hrvoje Buljan, John D. Joannopoulos, Marin Soljačić

**Affiliations:** 1 Department of Physics, Massachusetts Institute of Technology, Cambridge, MA 02139, USA; 2 Department of Applied Physics, Yale University, New Haven, CT 06520, USA; 3 Research Laboratory of Electronics, Massachusetts Institute of Technology, Cambridge, MA 02139, USA; 4 Physics Department and Solid State Institute, Technion, Haifa 32000, Israel; 5 Department of Physics, University of Zagreb, Zagreb 10000, Croatia

## Abstract

A bound state in the continuum (BIC) is an unusual localized state that is embedded in a continuum of extended states. Here, we present the general condition for BICs to arise from wave equation separability. Then we show that by exploiting perturbations of certain symmetry such BICs can be turned into resonances that radiate with a tailorable directionality and dimensionality. Using this general framework, we construct new examples of separable BICs and resonances that can exist in optical potentials for ultracold atoms, photonic systems, and systems described by tight binding. Such resonances with easily reconfigurable radiation allow for applications such as the storage and release of waves at a controllable rate and direction, as well systems that switch between different dimensions of confinement.

In most wave systems which support bound states, the bound states do not exist at the same frequency as the delocalized waves. However, there exist special systems for which a bound state can be embedded inside a continuum of delocalized waves^1^. Such bound states in the continuum (BICs) do not forbid coexistence with propagating waves at the same frequency, unlike traditional methods of localizing waves such as conventional potential wells in quantum mechanics, conducting mirrors in optics, band-gaps in periodic systems, and Anderson localization. BICs were first predicted theoretically in quantum mechanics by von Neumann and Wigner^2^. However, their BIC-supporting potential was highly oscillatory and could not be implemented in reality. More recently, other examples of BICs have been proposed theoretically in quantum mechanics^3^,^4^,^5^,^6^,^7^,^8^,^9^,^10^, electromagnetism^11^,^12^,^13^,^14^,^15^,^16^,^17^,^18^,^19^,^20^,^21^,^22^,^23^,^24^, acoustics^17^, and water waves^25^, with some experimentally realized^26^,^27^,^28^,^29^,^30^,^31^. A number of mechanisms explain most examples of BICs that have been discovered^1^.

Among them, BICs due to separability^4^,^5^,^6^,^7^,^12^,^13^,^14^,^15^ remain relatively unexplored with a scattered literature. In this paper, we develop the most general properties of separable BICs by providing the general criteria for their existence and by characterizing the continuua of these BIC-supporting systems. By doing so, we reveal properties that may lead to novel applications. In particular, our key result is that separable BICs enable control over both the directionality and the dimensionality of resonantly emitted waves by exploiting symmetry, which is not possible in other classes of BICs. Our findings lead to applications such as switchable-*Q* directional resonators and quantum systems which can be switched between quantum dot (0D), quantum wire (1D), and quantum well (2D) modes of operation. We provide experimentally realizable examples of separable BICs in photonic systems with directionally radiating resonances, as well as examples in cold atoms. The construction of these examples has two purposes. The first is to show that it is possible to construct experimentally realistic examples of separable BICs. The second being to guide experiments in the direction of realizing platforms to control the directionality and dimensionality of radiation.

## Results

### General Condition for Separable BICs

For instructional purposes, we start by reviewing a simple example of a two-dimensional separable system - one where the Hamiltonian operator can be written as a sum of Hamiltonians that each act on distinct variables *x* or *y*, i.e;


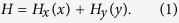


**Figure 1:**
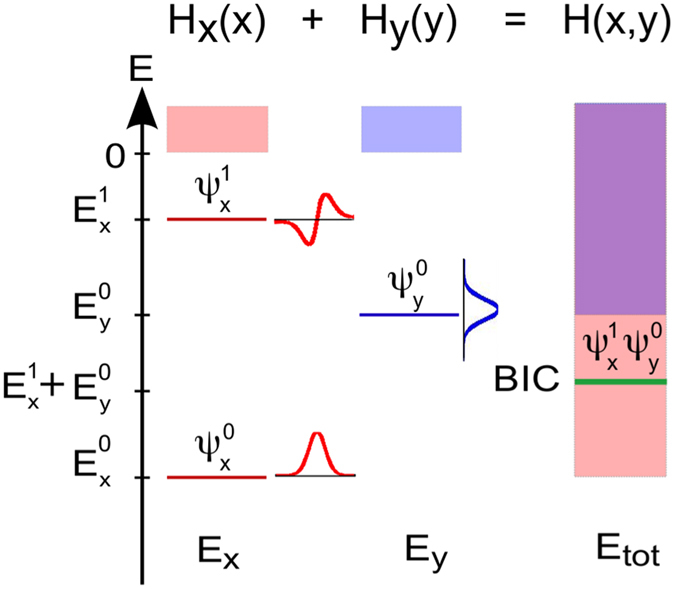
A schematic illustration demonstrating the concept of a separable BIC in two dimensions.

Denoting the eigenstates of *H*_*x*_ and *H*_*y*_ as 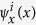 and 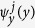, with energies 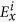 and 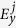, it follows that *H* is diagonalized by the basis of product states 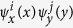 with energies 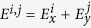. If *H*_*x*_ and *H*_*y*_ each have a continuum of extended states starting at zero energy, and these Hamiltonians each have at least one bound state (with negative energy), 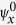 and 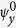, respectively, then the continuum of *H* starts at energy 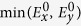, where the 0 subscript denotes ground states. Therefore, if there exists a bound state satisfying 
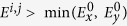
, then it is a bound state in the continuum. We schematically illustrate this condition being satisfied in Fig. 1, where we illustrate a separable system in which *H*_*x*_ has two bound states, 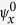 and 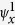, and *H*_*y*_ has one bound state, 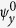. The first excited state of *H*_*x*_ combined with the ground state of *H*_*y*_, 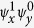, is spatially bounded in both x and y but has a larger energy, 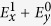, than the lowest continuum energy, 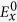, and is therefore a BIC.

Now, we extend separability to a Hamiltonian with a larger number of separable degrees of freedom. The Hamiltonian can be expressed in tensor product notation as 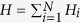, where *H*_*i*_ ≡ *I*^⊗*i*−1^ ⊗ *h*_*i*_ ⊗ *I*^⊗*N*−*i*^. In this expression, *N* is the number of separated degrees of freedom, *h*_*i*_ is the operator acting on the *i*-th degree of freedom, and *I* is the identity operator. The degrees of freedom may refer to the particle degree of freedom in a non-interacting multi-particle system, or the spatial and polarization degrees of freedom of a single-particle system. In any case, denote the *n*_*j*_th eigenstate of *h*_*i*_ by 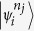 with energy 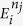. Then, the overall Hamiltonian *H* is trivially diagonalized by the product states 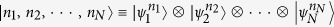 with corresponding energies 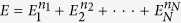. Denoting the ground state of *h*_*i*_ by 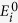 and defining the zeros of the *h*_*i*_ such that their continuua of extended states start at energy zero, the continuum of the overall Hamiltonian starts at 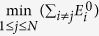. Then, if the separated operators {*h*_*i*_} are such that there exists a combination of separated bound states satisfying


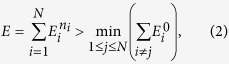


this combined bound state is a BIC of the overall Hamiltonian. For such separable BICs, coupling to the continuum is forbidden by the separability of the Hamiltonian. By writing the most general criteria for separable BICs, we can extend the handful of examples of separable BICs^4^,^5^,^6^,^7^,^12^,^13^,^14^,^15^ to systems in three dimensions, multi-particle systems, and systems described by the tight-binding approximation, allowing for a systematic way to generate realistic physical systems supporting BICs. While we only consider potentials separable in Cartesian and cylindrical coordinate systems, there are many more coordinate systems in which the Schrodinger equation can be separated^32^. Advances in the generation of complicated potentials using light should allow for the investigation of separable systems in more unconventional coordinate systems to be more than a mere mathematical curiosity.

### Properties of the Degenerate Continuua

A unique property that holds for all separable BICs is that the delocalized modes degenerate to the BIC are always trapped in at least one direction (and guided along the others). In many cases, there are multiple degenerate delocalized modes that are guided in different directions. When a system supporting a BIC is perturbed, this BIC generally turns into a resonance with finite lifetime. For 2D separable BICs which are perturbed into resonances, we can associate partial widths Γ_*x*_ and Γ_*y*_ to these resonances. These partial widths are the rates of coupling of the BIC to the radiation continuum (i.e., far-field) in the *x* and *y* directions under perturbation, respectively. When separability is broken, we generally can not decouple leakage in the *x* and *y* directions because the purely-*x* and purely-*y* delocalized continuum states mix. In this section, we show that one can control the radiation to be towards the x (or y) directions only, by exploiting the symmetry of the perturbation. We emphasize that although symmetry is used to realize control over directionality of radiation, separability is still generally required because the effect that we demonstrate has as the prerequisite a BIC degenerate to guided modes in different (separable) directions. As mentioned above, this is a unique property that holds for all separable BICs.

**Figure 2:**
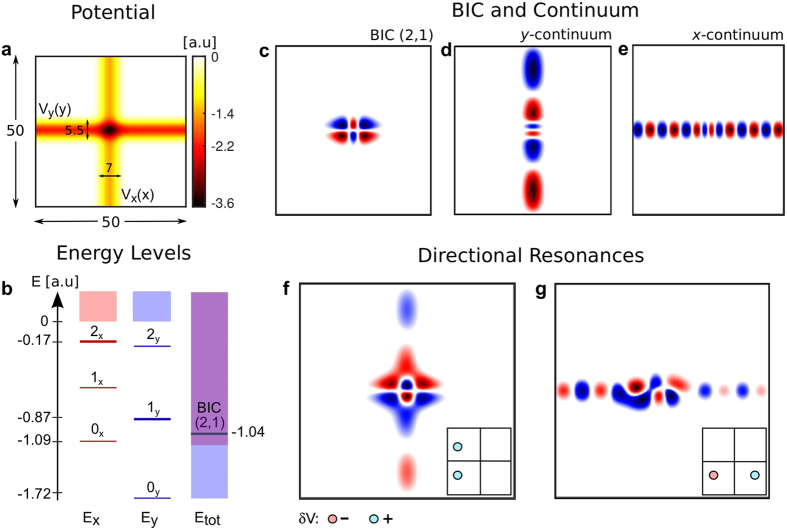
(**a**) A separable potential which is a sum of a purely x-dependent Gaussian well and a purely y-dependent Gaussian well. (**b**) The relevant states of the spectrum of the *x*-potential, *y*-potential, and total potential. (**c**) A BIC supported by this double well. (**d,e**) Continuum states degenerate in energy to the BIC. (**f**) A *y*-delocalized continuum state resulting from an even-*y*-parity perturbation of the BIC supporting potential. (**g**) An *x*-delocalized continuum state resulting from an odd-*x*-parity perturbation.

In Fig. 2(a), we show a two-dimensional potential in a Schrodinger-like equation which supports a separable BIC. This potential is a sum of Gaussian wells in the *x* and *y*-directions, given by


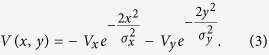


This type of potential may be realized as an optical potential for ultracold atoms or a refractive index contrast profile in photonic systems. In what follows in this section, we choose arbitrary values for the parameters in the potential in Equation (3). The effects that we report can of course be observed for many other parameter choices. Solving the time-independent Schrodinger equation for {*V*_*x*_, *V*_*y*_} = {1.4, 2.2} and {*σ*_*x*_, *σ*_*y*_} = {5, 4} (in arbitrary units) gives the energy spectra shown in Fig. 2(b). This system has several BICs. Due to the *x* and *y* mirror symmetries of the system, the modes have either even or odd parity in both the *x* and the *y* directions. Here we focus on the BIC |*n*_*x*_, *n*_*y*_〉 = |2, 1〉 at energy *E*^2,1^ = −1.04, with the mode profile shown in Fig. 2(c); being the second excited state in *x* and the first excited state in *y*, this BIC is even in *x* and odd in *y*. It is only degenerate to continuum modes 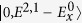 extended in the *y* direction (Fig. 2(d)), and 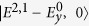 extended in the *x* direction (Fig. 2(e)), where an *E* label inside a ket denotes an extended state with energy *E*.

If we choose a perturbation *δV* that preserves the mirror symmetry in the *y*-direction, as shown in the inset of Fig. 2(f), then the perturbed system still exhibits mirror symmetry in *y* but not in *x*. Since the BIC |2, 1〉 is odd in *y* and yet the *x*-delocalized continuum states 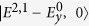 are even in *y*, there is no coupling between the two. As a result, the perturbed state radiates only in the *y* direction, as shown in the calculated mode profile in Fig. 2(f). This directional coupling is a result of symmetry, and so it holds for arbitrary perturbation strengths (within perturbation theory).

On the other hand, if we apply a perturbation that is odd in *x* but not even in *y*, as shown in the inset of Fig. 2(g), then there is radiation in the *x* direction only to first-order in time-dependent perturbation theory. Specifically, for weak perturbations of the Hamiltonian, *δV*, the first-order leakage rate is given by Fermi’s Golden Rule for bound-to-continuum coupling, 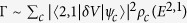, where *ρ*_*c*_(*E*) is the density of states of continuum *c*, and *c* labels the distinct continuua which have states at the same energy as the BIC. Since the BIC and the *y*-delocalized continuum states 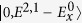 are both even in *x*, the odd-in-*x* perturbation does not couple the two modes directly, and Γ_*y*_ is zero to the first order. As a result, the perturbed state radiates only in the *x* direction, as shown in Fig. 2(g). At the second order in time-dependent perturbation theory, the BIC can make transitions to intermediate states *k* at any energy, and thus the second-order transition rate, proportional to 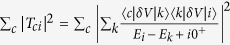, does not vanish because the intermediate state can have even parity in the *x*-direction. We note that although this argument relies on time-dependent perturbation theory, it is valid for any wave system provided that time evolution of the system is governed by a unitary operator and that the eigenstates of the unperturbed system form a complete basis. This is the case in the Schrodinger equation and in Maxwell’s equations for lossless dielectrics.

Another unique aspect of separability that we report here is that by using separable BICs in 3D, the number of confined dimensions of a wave can be switched between one, two, and three by tailoring perturbations applied to a single BIC mode. The ability to do this allows for a device which can simultaneously act as a quantum well, a quantum wire, and a quantum dot. We demonstrate this degree of control using a separable potential generated by the sum of three Gaussian wells (in *x, y*, and *z* directions) of the form in Equation 2 with strengths {0.4, 0.4, 1} and widths {12, 12, 3}, all in arbitrary units. Because of the separability in three dimensions, there are many more ways to combine bound and delocalized states in each dimension. For example, we can have a wavefunction bound in two dimensions and delocalized in the third, making a “wire” radiation pattern. We can also have a wavefunction bound in one dimension and delocalized in the other two, making a “sheet” radiation pattern. We can of course also make a wavefunction bound in all three directions, making a cavity mode. Importantly, we show via this example that all three of these types of modes can be made degenerate to each other.

The identical *x* and *y* potentials have four bound states at energies *E*_*x*_ = *E*_*y*_ = −0.33, −0.20, −0.10 and −0.029. The *z* potential has two bound states at energies *E*_*z*_ = −0.61 and −0.059. The BIC state |1, 1, 1〉 at energy *E*^1,1,1^ = −0.47 is degenerate to twelve continuum channels: |0, 0, *E*_*z*_〉, |0, 1, *E*_*z*_〉, |1, 0, *E*_*z*_〉, |*E*_*x*_, *m*, 0〉, |*n, E*_*y*_, 0〉, and |*E*_*x*_, *E*_*y*_, 0〉, where *E*_*i*_ denotes an energy above zero, and *m* ∈ {0, 1, 2, 3} and *n* ∈ {0, 1, 2, 3} denote bound states of the *x* and *y* wells. For perturbations which are even in the *z* direction, the BIC |1, 1, 1〉 does not couple to states delocalized in *x* and *y* (|*E*_*x*_, *m*, 0〉, |*n, E*_*y*_, 0〉, and |*E*_*x*_, *E*_*y*_, 0〉), because they have opposite parity in *z*, so the BIC radiates in the *z*-direction only. The resulting state is thus confined in two dimensions, as opposed to the BIC, which is confined in three. On the other hand, for perturbations which are even in the *x* and *y* directions, the coupling to states delocalized in *z* (|0, 0, *E*_*z*_〉, |0, 1, *E*_*z*_〉, and |1, 0, *E*_*z*_〉) vanishes, meaning that the BIC radiates in the *xy*-plane. This resulting state is confined in only in one dimension. Therefore, by tailoring perturbations to the potential, the number of confined dimensions of a wave can be switched between three, two, and one.

We now demonstrate one final general example which will serve not only to show another system in which the dimensionality of radiation can be controlled but also show that the concept of separable BICs transcends decomposition of the potential in Cartesian coordinates. Consider the following potential expressed in cylindrical coordinates *V*(*ρ, ϕ, z*) = *U*(*ρ*) + *W*(*z*), where *U*(*ρ*) = −*U*_0_ if *ρ* < *a* (and zero otherwise) and *W*(*z*) = −*W*_0_ if |*z*| < *b* (and zero otherwise). This is a cylindrical generalization of the separable sum of finite square well potentials in Cartesian coordinates. We can find the eigenvalues analytically for this potential. The eigenvalues of the radial well have two indices: *n* and *m*, where *n* is the excitation number and *m* is the angular index (the azimuthal dependence of the wavefunctions is *e*^*imϕ*^.) In the example which follows, the quantum number of the z-eigenfunctions will be labeled *n*_*z*_ and the three quantum numbers of the bound wavefunctions will be labeled |*n, m, n*_*z*_〉. The re-scaled Schrodinger equation reads:


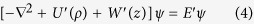


where primed variables denote unprimed quantities times 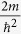 (the primed quanties have units of inverse length squared.) Taking *U*_0_ = 10, *a* = 1.6, *W*_0_ = 10, and *b* = 0.55 (all in arb. units, just as before), we find that the radial potential supports four bound states: |*n, m*〉 = |0, 0〉, |0, 1〉, |0, 2〉 and |1, 0〉. Their energies are −8.4, −6.2, −3.2, and −2.3 (respectively). For the z-dependent (axial) potential, there are merely two bound states |*n*_*z*_〉 = |0〉 and |1〉 at energies −6.8 and −0.25 respectively. Therefore, we can construct a BIC at energy −6.45 which is |0, 1, 1〉 (or the degenerate |0, −1, 1〉; we can take linear combinations of these two to form 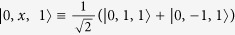 or 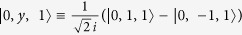). It is embedded in two continuua, associated either with the ground state of the radial well or the axial well. We can eliminate the coupling to the channel associated with the radial well by using a perturbation that is radially symmetric (but has no fixed parity in *z*). The resulting radiation is delocalized in *x* and *y* (it is a quantum-well like state). We can eliminate the coupling to the channel associated with the axial well by using a perturbation even in *z* (with no particular symmetry properties in *x* or *y*). The resulting radiation is delocalized in *z* (it is a quantum-wire like state).

### Proposals for the experimental realizations of separable BICs

BICs are generally difficult to experimentally realize because they are fragile under perturbations of system parameters. On the other hand, separable BICs are straightforward to construct and also robust with respect to changes in parameters that preserve the separability of the system. In the next two examples of BICs that we propose, we use detuned light sheets to generate separable potentials in photonic systems and for ultracold atoms.

#### Paraxial optical systems

As a first example of this, consider electromagnetic waves propagating paraxially along the *z*-direction, in an optical medium with spatially non-uniform index of refraction *n*(*x, y*) = *n*_0_ + *δn*(*x, y*), where *n*_0_ is the constant background index of refraction and *δn* ≪ *n*_0_. The slowly varying amplitude of the electric field *ψ*(*x, y, z*) satisfies the two-dimensional Schrödinger equation (see ref. 33 and references therein),


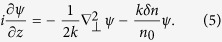


Here, 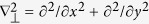, and *k* = 2*πn*_0_/*λ*, where *λ* is the wavelength in vacuum. The modes of the potential *δn*(*x, y*) are of the form 
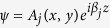
, where *A*_*j*_ is the profile, and *β*_*j*_ the propagation constant of the *j*th mode. In the simulations we use *n*_0_ = 2.3, and *λ* = 485 nm.

**Figure 3:**
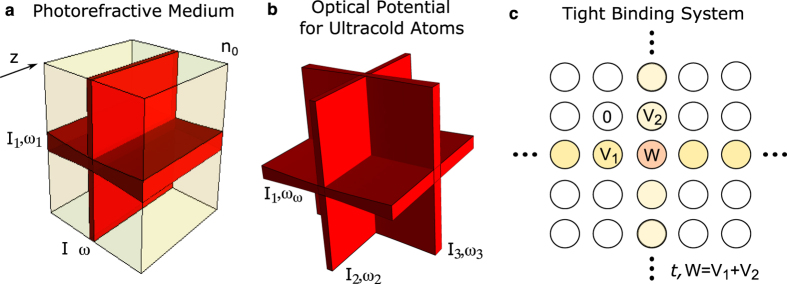
Separable physical systems with BICs. (**a**) A photorefractive optical crystal whose index is weakly modified by two detuned intersecting light sheets with different intensities. (**b**) An optical potential formed by the intersection of three slightly detuned light sheets with different intensities. (**c**) A tight-binding lattice.

Experimentally, there are several ways of producing systems modeled by Equation (4) for some *δn*(*x, y*) (e.g., periodic^34^, random^33^, and quasicrystal^35^, and others). One of the very useful techniques is the optical induction technique where the potential is generated in a photosensitive material (e.g., photorefractives) by laser writing^36^,^37^,^38^. We consider here a potential generated by laser writing using two perpendicular light sheets, which are slightly detuned in frequency, such that the time-averaged interference vanishes and the total intensity is the sum of the intensities of the individual light sheets. The light sheets are much narrower in one dimension (*x* for one, *y* for the other) than the other two, and therefore each light sheet can be approximated as having an intensity that depends only on one coordinate, making the index contrast separable. This is schematically illustrated in Fig. 3(a). If the sheets are Gaussian along the narrow dimension, then the potential is of the form *δn*(*x, y*) = *−δn*_0_[exp(−2(*x*/*σ*)^2^) + exp(−2(*y*/*σ*)^2^)]. It is reasonable to use *σ* = 30 *μ*m and *δn*_0_ = 5.7 × 10^−4^. For these parameters, the one-dimensional Gaussian wells have four bound states, with *β* values of (in mm^−1^): 2.1, 1.3, 0.55, and 0.11. There are eight BICs: |1, 2〉, |2, 1〉, |1, 3〉, |3, 1〉, |2, 3〉, |3, 2〉, |2, 2〉, and |3, 3〉. Among them, |1, 3〉 and |3, 1〉 are symmetry protected. Additionally, the BICs |1, 3〉 and |3, 1〉 can be used to demonstrate directional resonance in the *x* or *y* directions, respectively, by applying perturbations even in *x* or *y*, respectively. Therefore, this photonic system serves as a platform to demonstrate both separable BICs and directional resonances.

#### Optical potentials for ultracold atoms

The next example that we consider can serve as a platform for the first experimental realization of BICs in quantum mechanics. Consider a non-interacting neutral Bose gas in an optical potential. Optical potentials are created by employing light sufficiently detuned from the resonance frequencies of the atom, where the scattering due to spontaneous emission can be neglected, and the atoms are approximated as moving in a conservative potential. As is well known experimentally, the macroscopic wavefunction of the system is then determined by solving the Schrödinger equation with a potential that is proportional to the intensity of the light^39^,^40^. As an explicit example, consider an ultracold Bose gas of ^87^*Rb* atoms. An optical potential is produced similarly to our paraxial example by three Gaussian light sheets with equal intensity *I*_1_ = *I*_2_ = *I*_3_, widths 20*μ*m, and wavelengths centered at *λ* = 1064 nm^41^. This is schematically illustrated in Fig. 3(b). The intensity is such that this potential has depth equal to ten times the recoil energy 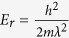. By solving the Schrodinger equation numerically, we find many BICs; the continuum energy starts at a reduced energy 
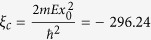
, where *x*_0_ is chosen to be 1 *μm*. The reduced depth of the trap is −446.93. Each one-dimensional Gaussian supports 138 bound states. There are very many BICs in such a system. For concreteness, an example of one is |30, 96, 96〉, with reduced energy −146.62. We conclude this example by noting that in the previous section, we described a way to change the dimensionality of radiation through special perturbations in a sum of three Gaussian wells, just like the sum of three Gaussian wells considered in this example. Therefore, we propose that a shallow optical potential for ultracold atoms can be used as a platform to control the dimensionality of radiation of a macroscopic atomic wave.

#### Tight Binding Models

The final example that we consider here is an extension of the separable BIC formalism to systems which are well-approximated by a tight-binding Hamiltonian that is separable. Such systems can be experimentally achieved, as in refs 37 and 38. Consider the following one-dimensional tight-binding Hamiltonian, *H*_*i*_, which models a one-dimensional lattice of non-identical sites (along direction i):


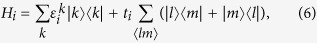


where 〈*lm*〉 denotes nearest-neighbors, 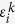 is the on-site energy of site *k*, and *k, l*, and *m* run from −∞ to ∞. Suppose 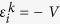 for |*k*| < *N*, and zero otherwise. For two Hamiltonians of this form, *H*_1_ and *H*_2_, *H* = *H*_1_ ⊗ *I* + *I* ⊗ *H*_2_ describes the lattice in Fig. 3(c). If we take *H*_1_ = *H*_2_ with {*V, t, N*} = {−1, −0.3, 2}, in arbitrary units, the bound state energies of the 1D-lattices are numerically determined to be −0.93, −0.74, −0.46, and −0.16. Therefore the states |2, 2〉, |2, 3〉, |3, 2〉, |3, 3〉, |3, 1〉 and |1, 3〉 are BICs. The last two of these are also symmetry-protected from the continuum as they are odd in *x* and *y* while the four degenerate continuum states are always even in at least one direction. Of course, many different physical systems can be adjusted to approximate the system from Eq. (4), so this opens a path for observing separable BICs in a wide variety of systems (coupled circuits, acoustic resonators, optical resonators, etc.).

### Summary

We have demonstrated two new properties unique to separable BICs: the ability to control the direction of emitted radiation using perturbations, and also the ability to control the dimensionality of the emitted radiation. This may lead to two applications. In the first, perturbations are used as a switch which can couple waves into a cavity, store them, and release them in a fixed direction. In the second, the number of dimensions of confinement of a wave can be switched between one, two, and three by exploiting perturbation parity. The property of dimensional and directional control of resonant radiation serves as a new and additional potential advantage of BICs over traditional methods of localization. Also, with the general criterion for separable BICs, we have extended the existing handful of examples to a wide variety of wave systems including: three-dimensional quantum mechanics, paraxial optics, and lattice models which can describe 2D waveguide arrays, quantum dot arrays, optical lattices, and solids. We conclude by pointing out how our findings generalize to the full vectorial Maxwell equations: in general, it will not be possible to find separable solutions to the full vectorial Maxwell equations because the Maxwell eigenoperator ∇ × ∇ × = ∇(∇⋅) − ∇^2^ isn’t separable due to the gradient term. In some systems, the gradient term vanishes, allowing for separable solutions. It may however be possible to find very high-Q resonance modes when the gradient term is weak.

## Additional Information

**How to cite this article**: Rivera, N. *et al*. Controlling Directionality and Dimensionality of Radiation by Perturbing Separable Bound States in the Continuum. *Sci. Rep.* **6**, 33394; doi: 10.1038/srep33394 (2016).
